# Effects of hypoxia-preconditioned HucMSCs on neovascularization and follicle survival in frozen/thawed human ovarian cortex transplanted to immunodeficient mice

**DOI:** 10.1186/s13287-022-03167-6

**Published:** 2022-09-14

**Authors:** Jiaojiao Cheng, Xiangyan Ruan, Yanglu Li, Juan Du, Fengyu Jin, Muqing Gu, Qi Zhou, Xin Xu, Yu Yang, Husheng Wang, Alfred Otto Mueck

**Affiliations:** 1 Department of Gynecological Endocrinology, Beijing Obstetrics and Gynecology Hospital, Capital Medical University, Beijing Maternal and Child Health Care Hospital, No. 251, Yaojiayuan Road, Chaoyang District, Beijing, 100026 People’s Republic of China; 2 Department for Women’s Health, University Women’s Hospital and Research Center for Women’s Health, University of Tuebingen, 72076 Tuebingen, Germany; 3 Department of Gynecology, Beijing Obstetrics and Gynecology Hospital, Capital Medical University, Beijing Maternal and Child Health Care Hospital, Beijing, 100026 People’s Republic of China

**Keywords:** Ovarian tissue transplantation, Human umbilical cord mesenchymal stem cell, PI3K/protein kinase B signalling pathway, Neovascularization, Follicle survival, Fertility preservation, Endocrine restoration, Hypoxia, Hypoxia-inducible factor 1α

## Abstract

**Background:**

The massive loss of follicles in the early stage of ovarian tissue transplantation is considered a significant restriction to the efficacy of ovarian tissue cryopreservation (OTC) and transplantation (OT). The use of mesenchymal stem cells (MSCs) before transplantation of ovarian fragments shortened the hypoxic period and boosted neovascularization. Hypoxia-preconditioned MSCs can enhance the potential of angiogenesis. Can hypoxia-preconditioned human umbilical cord mesenchymal stem cell (HucMSCs) and ovarian tissue co-xenotransplantation improve more neovascularization and subsequently more follicle survival in human ovarian tissue?

**Methods:**

Frozen-thawed cortical pieces from 4 patients were transplanted into the bilateral renal capsule of immune-deficient nude mice without HucMSCs or normoxia/hypoxia-preconditioned HucMSCs. Sixty-four mice were randomly distributed into 4 groups. In each group, the mice were euthanized for blood and/or graft retrieval on post-transplantation days 3 (*n* = 8) and 7 (*n* = 8), respectively. Non-grafted frozen-thawed ovarian fragment was taken for non-grafted control. Grafts were histologically processed and analysed for follicle density and atretic follicles by HE, neovascularization by CD34 and CD31 immunohistochemical staining, primordial follicle growth by Ki67 staining, and apoptosis of stromal cell and follicles by immunofluorescence using TUNEL. The ROS and TAC levels of grafted and non-grafted tissue were assessed. We evaluated the protein expression of HIF1α, VEGFA, pAkt, Akt, and GDF9 in grafted and non-grafted ovarian tissue. E2, Prog, AMH, and FSH levels in the plasma of mice were measured after 3 and 7 days of OT.

**Results:**

Hypoxia-preconditioned HucMSCs positively protect the grafted ovarian tissue by significantly decreasing the apoptosis and increasing higher expression of CD31, CD34, and VEGFA for earlier angiogenesis. They are crucial to preserving the resting primordial follicle pool by modulation of follicle death.

**Conclusion:**

This is the first study to demonstrate that co-transplantation of hypoxia-preconditioned HucMSC with ovarian tissue improved earlier vascularization of ovarian grafts in the early post-grafting period, which correlates with increased follicle survival and reduced apoptosis. The HIF1α/VEGFA signal pathways may play an important role in elucidating the mechanisms of action of hypoxia-preconditioned HucMSCs with regard to OT and clinical implementation.

## Introduction

Ovarian tissue cryopreservation (OTC) followed by transplantation (OT) is an established treatment for young girls and women with a high risk of premature ovarian insufficiency (POI) after toxic gonadal treatment such as chemotherapy, radiotherapy, or haematologic diseases requiring bone marrow transplantation [1, 2]. Ovarian function is recovered in 95% of women after OT, with pregnancy rates of about 40%, and more than 200 babies have been born worldwide as a result of this technique [3–6]. In 2019, The American Society for Reproductive Medicine (ASRM) considered the technique to be no longer experimental [7].

The lifespan of the ovarian graft depends on the number of primordial follicles, which is influenced by age and ovarian reserve at OTC. Unfortunately, this avascular tissue transplantation procedure without reanastomosis leads to follicle loss [5, 8]. Angiogenesis starts on day 3–5 after OT, whereas complete blood perfusion is achieved on day 6–10 [9, 10]. Experimental studies of OT generally show a 50% to 90% reduction in follicle density with undesirable follicle activation [11]. Despite the high re-establishment rate of endocrine function after OT, the follicular loss induced by hypoxia and ischaemia during the early stage of OT is of significant concern. It is directly related to graft longevity [2], and follicle survival in grafted tissues depends on the duration of hypoxia and the tissue response to promote revascularization. Many strategies have been implemented to improve follicle outcomes after OT by acting on the neovascularization of ovarian grafts. Among them, administration of growth factors, such as vascular endothelial growth factor (*VEGF*) [12], antioxidants, such as *N*-acetylcysteine (NAC) [13], hormones such as erythropoietin [14], and angiogenesis modulators such as sphingosine-1-phosphate (S1P) [15] have shown partial beneficial effects on follicle survival [16].

Over the past few years, mesenchymal stem cells (MSCs) have received much attention due to their significant therapeutic potential. MSCs can be attained from the umbilical cord, adipose tissue, bone, the placenta, etc. [17, 18]. Adipose tissue-derived stem cells (ASCs) have been employed to contribute to angiogenesis because ASCs differentiate into endothelial cells and improve the primordial follicles' survival in grafted ovarian tissue in a two-step operational procedure using a mouse model [16, 19]. However, methods aiming at treating the ovarian cortex at the transplantation site are highly desired to reduce follicle loss and burden to the patient. Human umbilical cord mesenchymal stem cells (HucMSCs), in contrast to other sources of MSCs, have become a prominent stem cell type for allogenic cell-based therapy because umbilical cords are easily isolated, their use is not affected by ethical arguments, low immunogenicity, or rapid renewal properties [20]. HucMSCs have been under investigation in various clinical therapeutic trials [20, 21].

The composition of secreted molecules varies significantly under different conditions, leading to differences in angiogenic capacities [22]. For example, it has been reported that under hypoxic conditions, the secretion of proangiogenic factors can be significantly increased [23]. Studies have shown that the beneficial effects of hypoxia on MSCs, including ASCs and HucMSCs, are mainly attributed to hypoxia-inducible factor 1α (*HIF1α*) [24]. Hypoxia upregulates *HIF1α* and enhances the viability, proliferation, secretion of soluble factors, chondrogenic potential, migration, and engraftment potential of MSCs [25, 26]. Preclinical experiments have shown that hypoxia-treated HucMSCs have an effective effect bone fracture healing, cartilage repair, and treating acute lung injury, indicating significant potential in treating ischaemic diseases and cartilage defects [27–29]. However, hypoxia-treated MSCs have not been subjected to ovarian tissue transplantation. This study aimed to evaluate the protective effect of hypoxia-preconditioned HucMSCs on the early stage of OT.

## Material and methods

### Experimental design

**Fig. 1:**
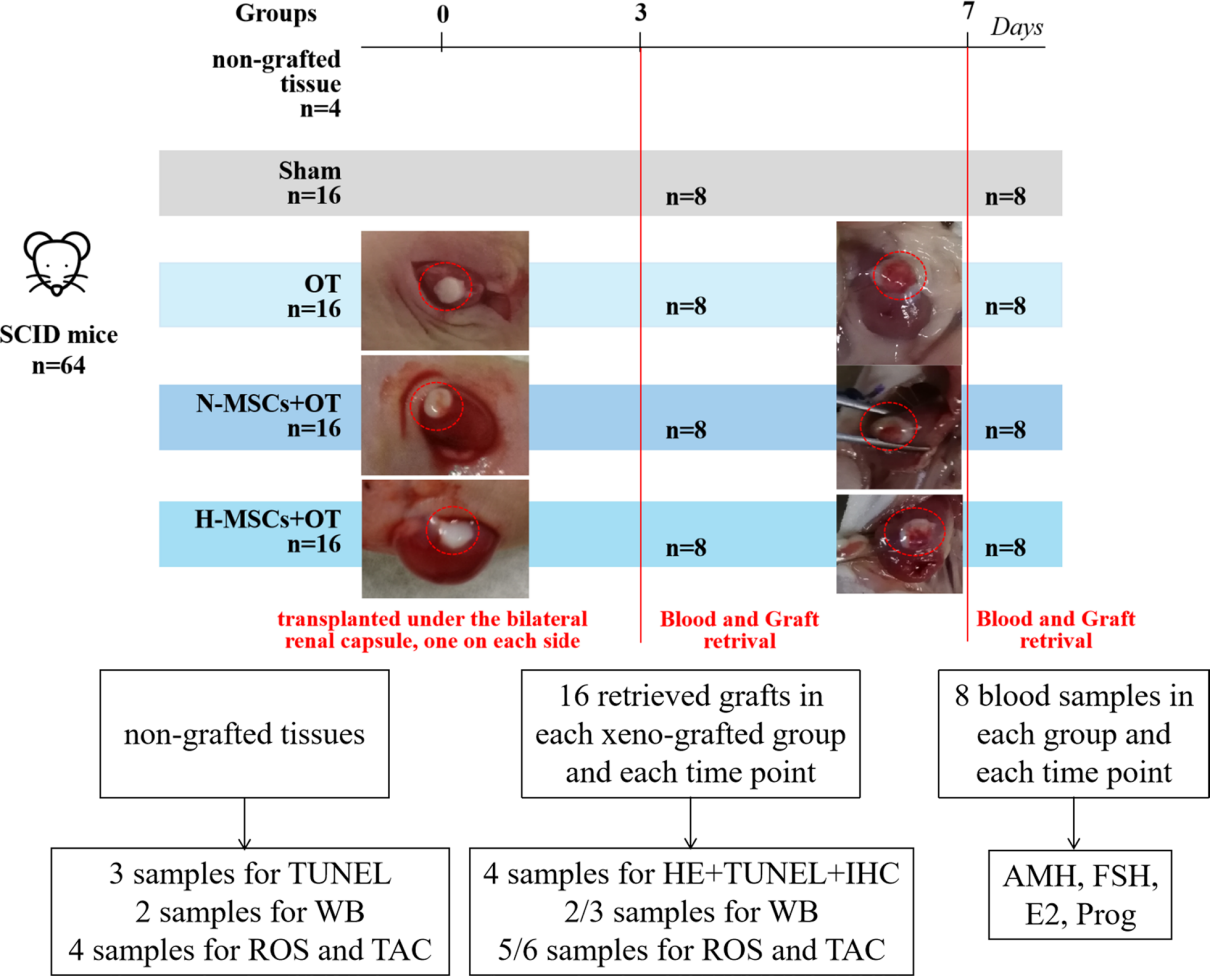
Experimental design. Frozen-thawed human ovarian cortex tissue from 4 patients was used in the experiment. Sixty-four mice were randomly distributed in 4 groups: (i) 16 mice undergoing OT without HucMSCs (OT group), (ii) 16 mice undergoing OT with 2 × 10^6^ normoxic-treated HucMSCs (N-MSCs + OT group), (iii) 16 mice undergoing OT with 2 × 10^6^ hypoxic-treated HucMSCs (H-MSCs + OT group), (iv) 16 mice undergoing bilateral oophorectomy but without OT (Sham-operated group). The ovarian cortex tissue from each patient (*n* = 4) was divided randomly into the OT, N-MSCs + OT, H-MSCs + OT groups, and non-grafted group. The SCID mice were euthanized for blood and graft retrieval on post-transplantation days 3 (*n* = 8) and 7 (*n* = 8), respectively. The ovarian tissue samples were evaluated by HE, TUNEL, IHC, WB, and ROS, TAC. The blood samples were assessed for E2, FSH, Prog, and AMH. Sham, sham-operated; OT, ovarian tissue transplantation; HucMSCs, human umbilical cord mesenchymal stem cells. N-MSCs + OT, normoxic-treated HucMSCs, and ovarian tissue co-transplantation. H-MSCs + OT, hypoxia-treated HucMSCs, and ovarian tissue co-transplantation; HE, haematoxylin and eosin; TUNEL, terminal deoxynucleotidyl transferase dUTP nick-end labelling; IHC, immunohistochemistry; WB, western blot; ROS, reactive oxygen species; TAC, total antioxidant capacity; E2, 17 β-estradiol; FSH, follicle-stimulating hormone; Prog, progesterone; AMH, anti-Müllerian hormone

Frozen-thawed human ovarian cortex tissue from 4 patients (age range 20–31 years) was used in the experiment. The ovarian tissue grafts consisted of a round cortex with a diameter of 3 mm, transplanted bilaterally under the renal capsules of nude mice. Sixty-four mice (Sibeifu Biotechnology Co., LTD., Beijing, License Number: SCXK (Beijing) 2019–0010) were randomly distributed into 4 groups: (i) 16 mice undergoing OT without HucMSCs (OT group), (ii) 16 mice undergoing OT with 2 × 10^6^ normoxic-treated HucMSCs (N-MSCs + OT group), (iii) 16 mice undergoing OT with 2 × 10^6^ hypoxic-treated HucMSCs (H-MSCs + OT group), (iv) 16 mice undergoing bilateral oophorectomy but without OT (Sham-operated group). The ovarian cortex tissue from each patient was divided randomly into the OT, N-MSCs + OT, and H-MSCs + OT groups. In each group, the mice were euthanized for blood and graft retrieval on post-transplantation days 3 (*n* = 8) and 7 (*n* = 8), respectively. One tissue fragment was taken directly from each patient as a non-grafted control (Fig. 1).

### Isolation and characterization of HucMSCs and preparation of normoxia and hypoxia-treated HucMSCs

HucMSCs were isolated and verified from the umbilical cords of healthy women after natural labour (three females, ages: 30, 33, and 25 years old) after obtaining informed consent according to institutional guidelines under the approved protocol [29]. Briefly, after removing the arteries and veins from the umbilical cord, the remaining tissue was cut into small pieces of 3–5 mm^3^, placed into the bottom of the culture bottle, cultured in Dulbecco’s Modified Eagles Medium–low glucose (Sigma-Aldrich, USA) supplemented with 10% foetal bovine serum (Sigma-Aldrich, USA) and 1% v/v penicillin–streptomycin (Gibco) and incubated at 37 °C and 5% CO_2_. One ml of culture medium was added every 24 h, and the medium was changed entirely after three days. The medium was subsequently changed twice a week. When the cells reached 70–80% confluency, they were digested with digestive juice containing 1.25ug/L trypsin (Life Technologies, Inc) and 1 g/L ethylenediaminetetraacetic acid (Life Technologies, Inc), then inoculated and subcultured at a density of 2.5–5.0 × 10^3^ /cm^2^. HucMSCs were identified by evaluating flow cytometry cell surface protein, CD73, CD90, and CD105 (> 95%), CD14, CD19, CD34, CD45, and HLA-DR (< 2%), which meets the particular surface antigen criteria to characterize MSCs.

HucMSCs were cultured and passaged at 37 °C in 5% CO_2_/95% air in StemPro™ MSC SFM medium (Gibco, Glasgow, Scotland), supplemented with 1% v/v L-glutamine (Gibco) and 1% v/v penicillin–streptomycin (Gibco). Cultures were washed in phosphate-buffered saline (PBS, Gibco) and passaged by enzymatic digestion using Accutase (Sigma-Aldrich, St. Louis, Missouri, USA) after achieving a density of 80–90%. Cells used in this study were all between passages 5 and 8.

HucMSCs were cultured until the number of cells needed for research was reached, and cultured HucMSCs were placed in a normoxic (21% O_2_, 37 °C) or hypoxic incubator (3% O_2_, 37 °C) for 48 h [22]. Hypoxia was achieved by maintaining cells in a modular incubator chamber (Billups-Rothenberg, Inc. Del Mar, CA, USA) flushed with a mixture of 3% O_2_, 5% CO_2_, 92% N_2_, confirmed by an infrared gas analyser (Novametrix Wallingford, CT, USA).

The normoxia or hypoxia-treated HucMSCs were centrifuged (500r/min, 5 min) and collected, resuspended in PBS (Life Technologies, Inc.), centrifuged again (500r/min, 5 min), organized, resuspended with precooled PBS and an equal volume of Matrigel^®^ hESC-Qualified Matrix (Cat. No. 354248; Corning^®^, Waltham, MA) and mixed evenly. The Matrigel + H-MSCs/ N-MSCs suspension was placed on ice before transplantation with ovarian tissue grafts. Matrigel is liquid at low temperatures and becomes solid at 37 °C, which means when Matrigel + HucMSCs is injected under the renal capsule of nude mice and around ovarian tissue grafts, it becomes immobile to reduce the loss of HucMSCs.

For identification, the normoxia or hypoxia-treated HucMSCs were trypsinized, washed with PBS, and reacted with APC-anti-CD90, APC-anti-CD44, APC- anti-CD14, APC- anti-HLA-DR, and APC- anti-CD45 (BD Biosciences, USA). After washing with PBS, the expression level of these molecules was determined by flow cytometry. The isotope antibodies were used as negative controls.

### Ovarian tissue cryopreservation and thawing procedure

The study's use of human ovarian tissue and umbilical cord tissue was approved by the Ethics Committee of the Beijing Obstetrics and Gynecology Hospital, Capital Medical University (No: 2020-KY-007-01, 2020/April 20). Frozen-thawed human ovarian tissue from 4 different patients (age range 20–31 years) was taken from our ovarian tissue cryobank and used for the study. All 4 women (breast cancer *n* = 4) had provided written informed consent to donate no more than 10% of cryopreserved ovarian tissue for research purposes. The number of follicles from the 4 patients was 146, 50, 45, 95/circular cortex 3 mm in diameter, respectively, evaluated by calcein-AM, as previously described [30]. Ovarian tissue was prepared with a circular diameter of 3 mm and frozen using the slow-freezing protocol, as previously described [30, 31]. Cryogenic vials were thawed at room temperature for 1 min, immersed in a water bath at 37 °C for 2 min, then sequentially placed in thawing media with decreasing sucrose contractions, as previously described [32].

### Surgical procedure

Guidelines for animal welfare were fully adhered to, and the study was approved by the Committee on Animal Research of the Capital Medical University (No: AEEI-2020–064, Date: May 6, 2020). Sixty-four 8-week-old female severe combined immunodeficient (SCID) mice (Sibeifu (Beijing) Biotechnology Co., LTD., License Number: SCXK (Beijing) 2019-0010) were used for this study. Mice were housed in groups, fed pellets, given water ad libitum, and kept under controlled 12 h light/ 12 h dark cycles at 20–22 °C. One week after arrival, the mice were ovariectomized. The mice were anesthetized by intraperitoneal injection of sodium pentobarbital (40 mg/kg, Sigma-Aldrich). Two weeks after ovariectomy, human frozen-thawed ovarian tissue was transplanted into immunodeficient mice housed on their own for 3 or 7 days. The experiment was divided into 4 groups: (i) OT group, (ii) N-MSCs + OT group, (iii) H-MSCs + OT group, (iv) Sham group undergoing ovariectomy but without OT.

After skin disinfection of the nude mice, the skin and muscle layer were cut in turn under the left costal arch, making a small oblique orifice about 1 cm and exposing the left kidney. The kidney was gently squeezed out of the abdominal cavity and clamped. The renal capsule was lifted, and a small hole was made in the renal capsule using the needle of a 50 ml syringe. The frozen-thawed human ovarian cortex was inserted under the completely stripped renal capsule using flat forceps without using sutures. In the H-MSCs + OT group and N-MSCs + OT group, the H-MSCs/ N-MSCs suspension of 20 μl (1 × 10^6^ MSCs, including 30% Matrigel) was injected around the subcapsular cortex after the ovarian cortex was transplanted into the renal capsule. The kidney was carefully placed back into the abdominal cavity, and the muscle layer and skin incisions were closed with absorbable 5/0 Prolene (Ethicon, Somerville, NJ, USA). The transplantation procedure on the right side was the same as the left side, and each mouse underwent the same treatment in both sites. The mice were kept in sterile conditions for the entire duration. Our study chose the ovarian grafts to be recovered after 3 or 7 days because 3–7 days after OT represents a critical window during graft re-vascularization [19].

### Graft retrieval and histological processing

Animals were sacrificed at days 3 and 7 post-grafting by anaesthesia with intraperitoneally administered Nembutal^®^, 100 mg/kg. The grafts were quickly identified, recovered, and cleaned to remove murine kidney tissue. There were 16 grafts in each group at each time point. Four xenografts from 4 patients were retrieved and fixed in 4% paraformaldehyde for follicles evaluation by stained with haematoxylin and eosin (HE) (Merck, Germany). They were analysed for vascularization by immunohistochemistry (IHC) using CD34 and CD31 staining, primordial follicle growth by Ki67 staining, and stroma cell and follicle apoptosis by terminal deoxynucleotidyl transferase dUTP nick-end labelling (TUNEL). All the above indicators were estimated in 4 different sections/slides of each graft. TUNEL was also evaluated in 3 non-grafted fragments. Grafts and non-grafted fragments were fixed overnight, dehydrated, and embedded in paraffin. Samples were cut into 5-μm-thick sections. The 4 slices from each index comprised 2 from near the medulla surface of the ovarian cortex and 2 from near the cortex surface, and the thickness difference between the same side two slices was about 50 μm. All paraffin-embedded ovarian tissue sections were heated for 25 min at 65 °C before HE, IHC, and TUNEL staining. For all downstream analyses, cortex slides were processed in parallel.

Other grafts were snap-frozen in liquid nitrogen-cooled isopentane and stored at -80 °C. In each group and each time point, there were 2 or 3 grafts from different patients for western blot (WB), 5 or 6 grafts from 4 patients for reactive oxygen species (ROS), and total antioxidant capacity (TAC) analysis. 17 β-estradiol (E2), follicle-stimulating hormone (FSH), progesterone (Prog), and anti-Müllerian hormone (AMH) levels in mice blood samples of different groups and time points were investigated.

### Histological and immunohistochemical staining

#### Follicle density and classification

Four grafted samples from each time point group were fixed in 4% formaldehyde, embedded in paraffin, and serially sectioned (5-μm-thick sections). Four slides were stained with HE for follicle evaluation. After staining and immunolabelling, all slides were digitized by automated whole-slide image capture using the Pannoramic P250 Flash III scanner (3DHISTECH, Hungary). Image files were analysed with CaseViewer (v2.3). Follicles with a clear oocyte surrounded by organized granulosa cells, an intact basal membrane, and a nucleus with dispersed chromatids were considered "healthy". Follicles with a condensed nucleus, reduced ooplasm and cytoplasm, and shrunken granulosa cells were regarded as "atretic" [33].

Only morphologically healthy follicles with an apparent nucleus (~ 18 μm in diameter) were counted and used for follicle density measurements. Four sections were counted, and the total number of follicles in the graft was evaluated and quantified. Follicle density was calculated based on total follicle count and graft volume. Healthy follicles were classified according to stage into resting follicles and growing follicles. The resting follicle pool includes primordial and intermediate follicles, characterized by oocytes surrounded by a single layer of flattened granulosa cells or a combination of flattened and cuboidal granulosa cells. Other follicles were considered growing, including primary and secondary follicles, respectively, characterized by oocytes surrounded by a single layer of cuboidal granulosa cells (GCs) or two or more layers of cuboidal GCs [13]. Two independent investigators who were blinded to the experimental treatments performed all analyses.

#### Follicle apoptosis

TUNEL staining was performed according to the manufacturer's instructions (DeadEndTM Fluorometric TUNEL System, Promega Corp., Madison, WI, USA). Apoptosis was evaluated in the three non-grafted tissues and the 4 grafts of each group and each time point. The details were the same as in our previous publication [34]. The tissue information from 4 slides from each non-grafted tissue and grafts of each group of each time point were scanned using a panoramic section scanner (PANORAMIC DESK/MIDI/250/1000; 3DHISTECH, Hungary). After the image scanning was completed, CellQuant software (3DHISTECH) automatically identified and set all the identical green, fluorescent nuclei on the tissue section as the unified standard for judging all TUNEL-positive cells. The identical 4′,6-diamidino-2-phenylindole (DAPI) blue nuclei were DAPI-positive cells. A series of data such as TUNEL-positive cells and DAPI-positive cells in each slice was automatically analysed using QuantCenter 2.1. The percentage of apoptotic stromal cells was calculated by dividing the number of TUNEL-positive cells by the number of DAPI-positive cells. All morphologically normal follicles were analysed. Follicles were considered positive if more than 50% of GCs or oocytes were stained [35].

### Immunohistochemical staining for graft vascularization and follicles growth

IHC was performed to detect CD31, endothelial cell markers, to visualize human vascularization of the xenograft human ovarian tissue at two time points and in three groups. CD34 IHC was performed to assess human and murine vascularization of the xenograft. Ki67 IHC was performed to evaluate the growth of primordial follicles. Four slides (5 μm) from each graft were used to detect each index. Paraffin sections were dewaxed to water, tris–EDTA antigen repair buffer solution (PH 9.0, G1203, Servicebio, China) was used for antigen retrieval, 3% hydrogen peroxide (H_2_O_2_) (G0115, Servicebio, China) was used to block endogenous peroxidase, and bovine serum albumin (BSA, G5001, Servicebio, China) was used as blocking serum. Sections were incubated at 4 °C overnight with the primary antibody, Ki67 antibody (GB111141, 1:800, Servicebio, China), CD34 antibody (GB13013, 1:500, Servicebio, China), CD31 antibody (GB13428, 1:500, Servicebio, China), and for 60 min at room temperature with the secondary antibody, horseradish peroxidase (HRP) goat anti-rabbit antibody (G1211, Servicebio, China). 3,3′-Diaminobenzidine (DAB) chromogenic solution was used for chromogenic development, haematoxylin was used to counterstain the nuclei, and the slides were dehydrated. The grafted human ovarian tissue slides were used as positive controls. For the negative controls, other grafted human ovarian tissue slides were incubated without primary antibodies to ensure the corresponding markers were not present. All samples were performed based on the manufacturer’s instructions. The tissue information from each slide was scanned using a panoramic section scanner (PANNORAMIC DESK/MIDI/250/1000; 3DHISTECH, Hungary). Scanning and browsing software was CaseViewer2.4 (3DHISTECH Hungary). Analysis software was Halo v3.0.311.314 (Indica Labs, USA).

Brown colouring of the cytoplasm/nucleus of the cells was specified as positive staining (anything else as negative staining). A positive cell staining assessment was performed in 4 different fields per slide at 200 × magnification [36]. Vascularization was calculated and expressed as the number of CD34-positive per section area or CD31-positive vessels per section area, in other words, vessel density.

Primordial follicle growth was evaluated and expressed as the ratio of Ki67-positive primordial follicles to the total number of primordial follicles. In terms of follicular growth, when all granulosa cells are Ki67 negative, the primordial follicles are considered quiescent. When at least one GC is Ki67 positive, the primordial follicles are considered growing [19, 37]. Two independent investigators who were blinded to the experimental treatments performed all measurements.

### ROS and TAC

ROS levels in the 5 or 6 grafts in each time point of each group and 4 non-grafted fragments were separately assessed by a spectrofluorometric method using 2′,7′-dihydro-dichlorofluorescein diacetate (DCHF-DA) assay, as previously described [38]. The TAC assay was also measured using the trolox equivalent antioxidant capacity method in these cortex samples, as previously described [38]. Briefly, this assay assesses the total radical scavenging capacity based on a compound's ability to scavenge the stable 2,2′-azino-bis-3-ethylbenzothiazoline-6-sulfonic diammonium salt radical within 3 min. All experiments were performed within three independent experiments.

### Western blot

*VEGFA*, *HIF1a*, growth differentiation factor 9 (*GDF9*), phosphor-protein kinase B (*p-Akt*), protein kinase B (*Akt*) protein expression in 2 or 3 grafts of each time point of each group and two non-grafted fragments were separately evaluated by western blot. The procedures were performed according to the previous description [34]. Ovarian tissue lysates were obtained using RIPA lysis, and extraction buffer (catalogue #89900; Thermo Fisher, USA) supplemented with protease inhibitor (catalogue #04693159001; Roche, Switzerland) and phosphatase inhibitor (catalogue #04906837001; Roche). The extracted protein concentration was measured according to the Bradford protocol. Equal amounts of total protein (50 μg) were boiled in loading dye for 10 min and subjected to 15% sodium dodecyl sulphate–polyacrylamide gel electrophoresis (SDS-PAGE). Protein bands were transferred to polyvinylidene fluoride (PVDF) membranes (Bio-Rad Laboratories, USA), and the membranes were blocked with 1 TBST (catalogue #TA-125-TT; Thermo Fisher, USA) containing 5% skimmed milk (catalogue #232100, BDbiosciences, USA) and 0.05% Tween-20 at room temperature for three hours. The membranes were probed with anti-*VEGFA* (1:1000 dilution, catalogue #214424; Abcam, USA), anti-*HIF1α* (1:1000 dilution, catalogue #179483; Abcam, USA), anti-*GDF9* (1:1000 dilution, catalogue #273455; Abcam, USA), anti-*p-Akt* (1:1000 dilution, catalogue #4060; Cell Signaling Technology, USA), anti-*Akt* (1:1000 dilution, catalogue #4691; Cell Signaling Technology, USA), and anti-β-actin (1:5000, catalogue #A5441; Sigma, USA) primary antibodies at 4 °C overnight with gentle shaking. Following incubation, the membranes were washed three times with 1 TBST and incubated with goat anti-rabbit secondary antibody (1:5000 dilution, catalogue #7074; Cell Signaling Technology, USA) in 1 TBST containing 3% skimmed milk at room temperature for 60 min. Immunoreactive proteins were visualized by chemiluminescence using ECL western blotting substrate (catalogue #34095, Thermo Fisher, USA) and detected on Tanon 4600 chemiluminescent imaging system (Agfa-Gevaert, Belgium). The protein expression was quantified using ImageJ software and expressed as the value relative to β-actin.

### Hormonal assays

Blood samples were collected during the day of graft retrieval after anaesthesia. The samples were held at room temperature for 15 min and centrifuged at 2000*g* for 20 min. The supernatant was collected and stored at − 80 °C. E2, FSH, Prog, and AMH were measured using the E2 enzyme-linked immunosorbent assay (ELISA) kit (ab108640, Abcam, China), FSH ELISA Kit (ab108678, Abcam, China), Prog ELISA Kit (ab108670, Abcam, China) and AMH ELISA Kit (R&D DY1737, Bio-Techne China Co. Ltd.). Each experiment was repeated three times.

### Statistical analysis

Results are expressed as mean ± standard error of the mean (SEM). Statistical analysis was performed using SPSS Statistics for Windows, version 23.0 (IBM Corp., Armonk, NY, USA) and GraphPad Prism 6.0 (GraphPad Software, Inc., CA, USA). Follicle data were analysed with a linear mixed-effects model with patient effect as a random effect due to the heterogeneous follicle distribution in the cortical tissue pieces within the same patient and between patients. One-way ANOVA or the Kruskal–Wallis test was used, depending on normality distribution, with Fisher's least significant difference (LSD) or uncorrected Dunn's test as a post hoc test. Results were presented as mean ± SEM. Only significant differences between different groups in one-time points and time points in one group and non-grafted groups with other groups were documented and discussed. *P < 0.05, **P < 0.01, and ***P < 0.001 were considered to be significant.

## Results

### Characterization of normoxia or hypoxia-treated HucMSCs

**Fig. 2:**
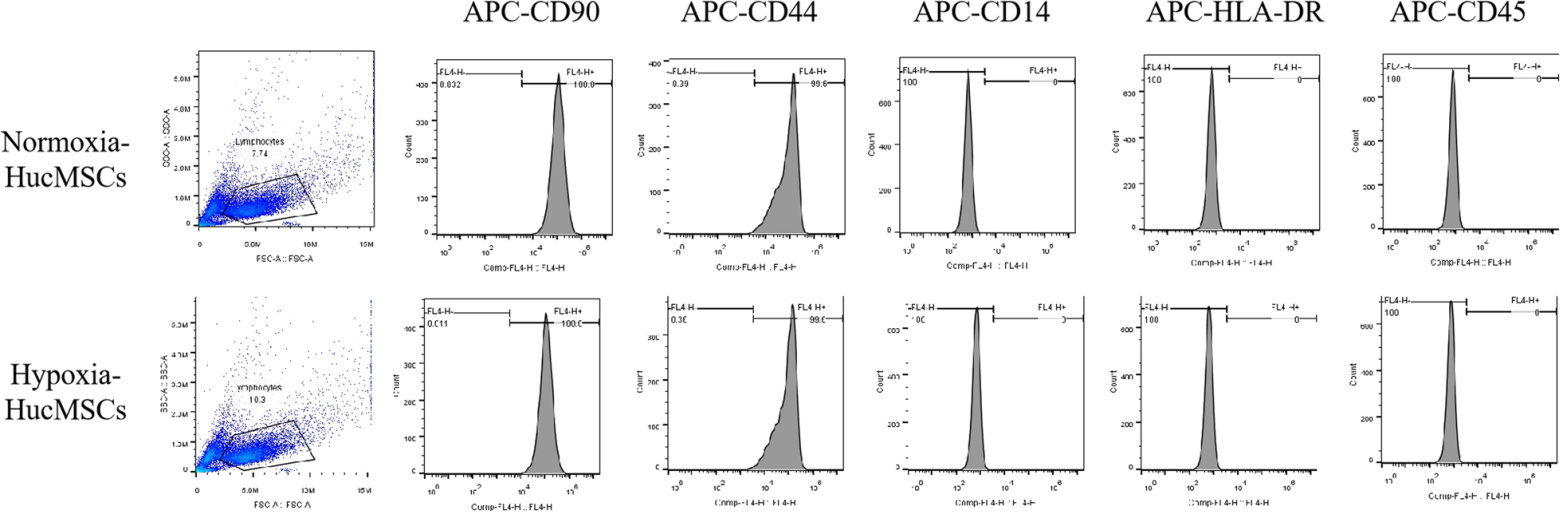
Detection of surface markers of normoxia or hypoxia-treated HucMSCs by flow cytometry. Flow cytometry cell surface protein profile positive for CD90, CD44 (> 99%), and negativity for CD14, HLA-DR, and CD45 (< 1%) in normoxia and hypoxia-treated HucMSCs

Flow cytometry cell surface protein profile was positive for CD90, CD44 (> 99%), and negative for CD14, HLA-DR, and CD45 (< 1%) in normoxia and hypoxia-treated HucMSCs, which meets the special surface antigen criteria to characterize MSCs (Fig. 2).

### Graft recovery rates and macroscopic assessment

All grafted ovarian tissues were retrieved from all 48 mice attached to their renal capsules. Apparent angiogenesis appeared in the grafts after seven days of transplantation (Fig. 1).

### Follicle counts and classification

**Fig. 3:**
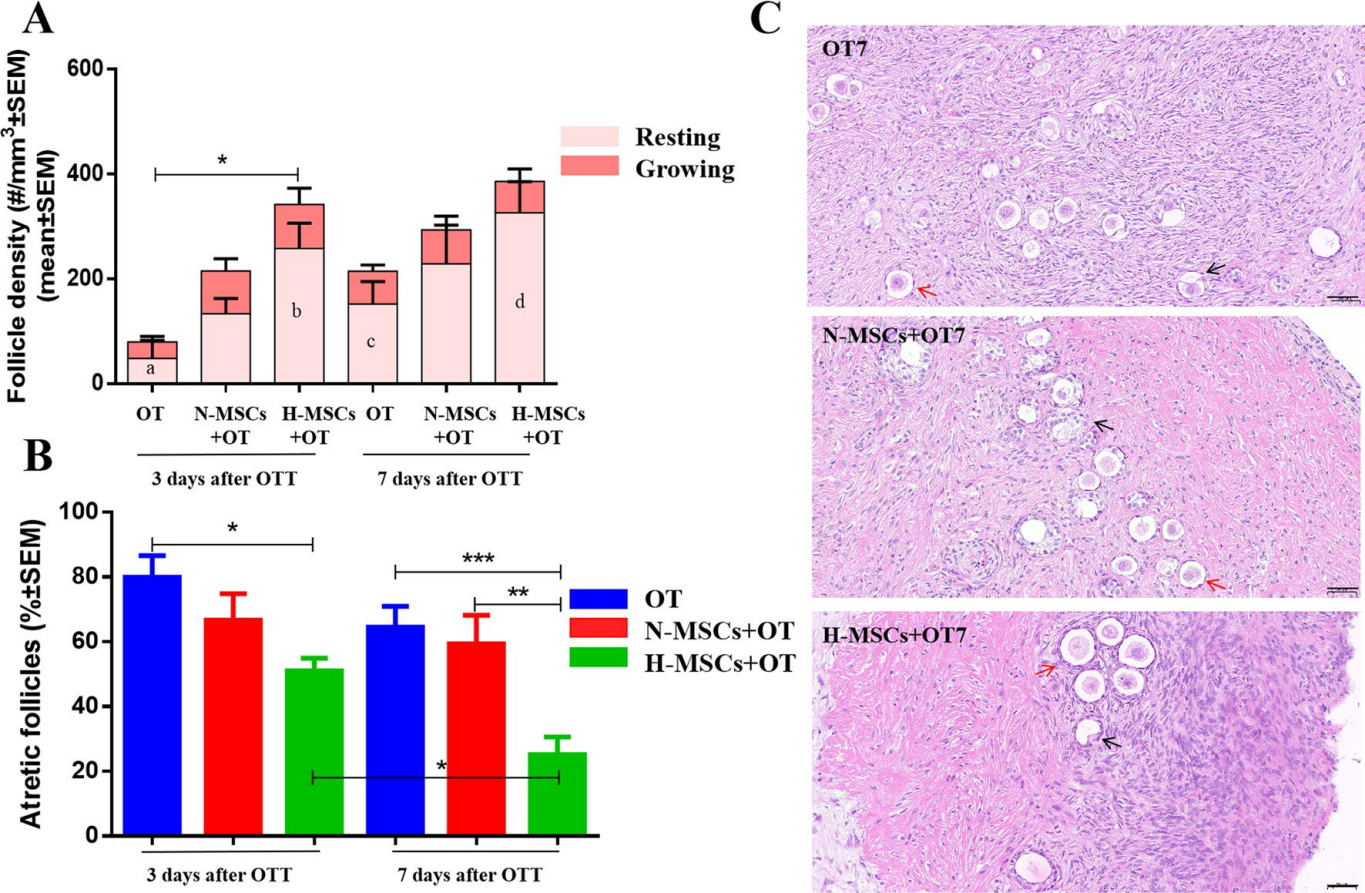
Follicle outcomes. **A** Follicle density (no. of follicles/mm^3^ ± SEM) in the general follicle population and at each stage (resting and growing) was evaluated by one-way ANOVA and Fisher’s LSD post hoc test. **B** Follicle atresia (% of atretic follicles/total follicles) was evaluated by the Kruskal–Wallis and Fisher’s post hoc tests. **C** HE staining of grafted tissues in the OT, N-MSCs + OT, and H-MSCs + OT groups on day 7, red arrows represent morphologically normal follicles (“healthy”), black arrows represent morphologically abnormal follicles (“atretic”). *P* value references: *P < 0.05; **P < 0.01; ***P < 0.001. Statistical significance is expressed in subcolumns using different letters, a vs. b *P* < 0.05, c vs. d *P* < 0.05, Resting follicles density was significantly higher in the H-MSCs + OT group than OT group on day 3 and day 7. Scale bars: 50 μm. OT, ovarian tissue transplantation; N-MSCs + OT, normoxic-treated HucMSCs, and ovarian tissue co-transplantation. H-MSCs + OT, hypoxia-treated HucMSCs, and ovarian tissue co-transplantation. All graphs show mean ± SEM and *n* = 4 per group and per time point

Follicle density and classification were evaluated in HE stained sections (Fig. 3). Follicle density (no. follicles/mm^3^ ± SEM) was significantly higher in the H-MSCs + OT (332.79 ± 69.66) group than OT (69.12 ± 39.67, vs. *P* = 0.037) group on day 3. Regarding follicle stages, densities of resting and growing follicles were investigated. Resting follicles density was significantly higher in the H-MSCs + OT (258.4 ± 48.14, 326.12 ± 59.67) group than OT (48.29 ± 34.61, vs. *P* = 0.036; 152.47 ± 42.95, vs. *P* = 0.028, respectively) group on day 3 and day 7.

Regarding percentages of atretic follicles, a significant decrease in atresia was detected in the H-MSCs + OT (51.07 ± 3.91) group on day 3 compared to the OT group (79.97 ± 6.66, vs. *P* = 0.023). A considerable reduction in atresia was seen in the H-MSCs + OT (25.11 ± 16.62) group on day 7 compared to the OT group (64.53 ± 6.41, vs. *P* < 0.001) and N-MSCs + OT group (59.35 ± 8.82, vs. *P* = 0.003). A significant decrease in atresia was detected in the H-MSCs + OT group on day 7 compared to day 3 (*P* = 0.020).

### Grafts stroma cells apoptosis

**Fig. 4:**
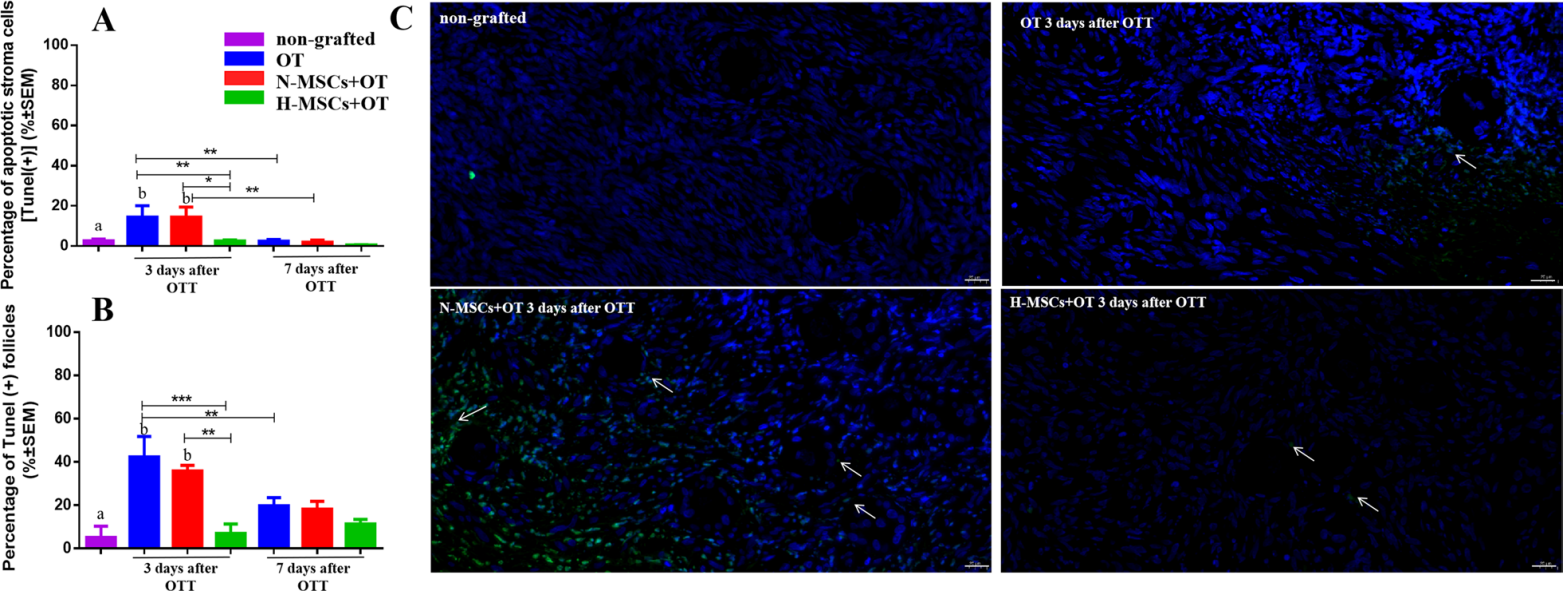
TUNEL-positive stroma cells and follicles. **A** percentage of TUNEL-positive apoptotic stroma cell, **B** percentage of TUNEL-positive apoptotic follicle, data were represented as mean ± SEM and analysed by one-way ANOVA and Fisher’s LSD post hoc test. **C** TUNEL staining of non-grafted and grafted tissues in the OT, N-MSCs + OT, and H-MSCs + OT groups on day 3. Green fluorescence: TUNEL-positive nuclei; blue fluorescence: DAPI. White arrows: TUNEL-positive. Scale bars: 50 μm. *P* value references: *P < 0.05; **P < 0.01; ***P < 0.001. Statistical significance is also expressed using different letters, a vs. b *P* < 0.05. TUNEL, terminal deoxynucleotidyl transferase dUTP nick-end labelling; OT, ovarian tissue transplantation; N-MSCs + OT, normoxic-treated HucMSCs, and ovarian tissue co-transplantation. H-MSCs + OT, hypoxia-treated HucMSCs, and ovarian tissue co-transplantation. *n* = 4 per group and per time point

The percentage of apoptotic stroma cells in the ovarian grafts was revealed by the TUNEL assay (Fig. 4A). The rate of TUNEL (+) stromal cells was significantly higher in the OT group and N-MSCs + OT after 3 days (14.46 ± 5.68, vs. *P* = 0.011; 14.47 ± 4.97, vs. *P* = 0.016) post-transplantation compared to non-grafted controls (2.75 ± 0.80). No significant increase was detected in the OT group and N-MSCs + OT after 7 days (2.56 ± 0.84, vs. *P* = 0.965; 2.06 ± 0.95, vs. *P* = 0.870). Meanwhile, no significant increase was seen in the H-MSCs + OT group (day 3: 2.64 ± 0.46; day 7: 0.66 ± 0.18). Significantly fewer apoptotic stroma cells were detected in the H-MSCs + OT than N-MSCs + OT and OT groups (*P* = 0.006; *P* = 0.010, respectively) after 3 days of OT.

### Follicle apoptosis

A total of 695 normal-looking follicles were analysed for TUNEL (+) marker: 43 from non-grafted controls, 103, 143, and 49 from the OT, N-MSCs + OT, and H-MSCs + OT groups on day 3, 110, 118, and 129 from the OT, N-MSCs + OT, and H-MSCs + OT groups on day 7. Percentage of TUNEL (+) follicles were significantly higher in the OT group and N-MSCs + OT on 3 days (42.36 ± 9.44, vs. *P* < 0.001; 35.90 ± 2.57, vs. *P* = 0.004) than in non-grafted control (5.13 ± 5.13). No significant difference was detected in the OT group and N-MSCs + OT on 7 days (19.79 ± 3.73, vs. *P* = 0.075; 18.18 ± 3.60, vs. *P* = 0.109) compared with the non-grafted control. Meanwhile, no significant difference was seen in the H-MSCs + OT group (Day 3: 6.93 ± 4.45, vs. *P* = 0.819; Day 7: 11.37 ± 2.09, vs. *P* = 0.431) compared with the non-grafted group. Notably, the percentage of TUNEL (+) follicles were significantly lower in the H-MSCs + OT group than the OT group and N-MSCs + OT (*P* < 0.001, *P* = 0.004, respectively) on day 3 (Fig. 4B).

### Grafts neovascularization

**Fig. 5:**
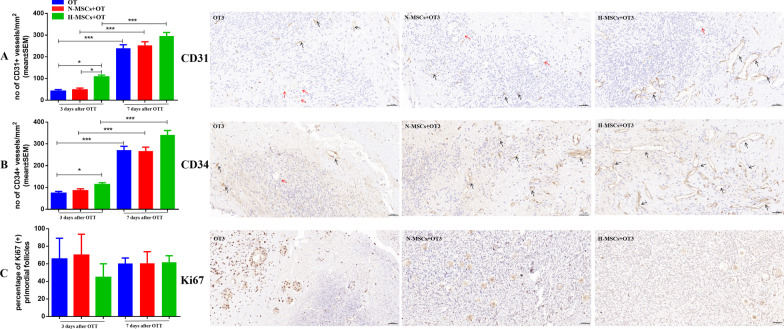
**A** Total endothelial area by CD31 immunohistochemistry to evaluate the percent of the vessel-covered area. **B** Total endothelial area by CD34 immunohistochemistry to evaluate the percent of the vessel-covered area. **C** The percentage of Ki67 (+) primordial follicles was evaluated in grafts. Histological features of human ovarian tissues in the grafts of ovarian tissue evaluated with immunohistochemistry staining for CD31, CD34, and Ki67. CD31 and CD34 were represented as mean ± SEM and analysed by one-way ANOVA and Fisher’s LSD post hoc test. Ki67 was represented as mean ± SEM and analysed by the Kruskal–Wallis and Fisher’s post hoc tests. Scale bars: 50 μm. *P* value references: *P < 0.05; ***P < 0.001. OT, ovarian tissue transplantation; N-MSCs + OT, normoxic-treated HucMSCs, and ovarian tissue co-transplantation. H-MSCs + OT, hypoxia-treated HucMSCs, and ovarian tissue co-transplantation. *n* = 4 per group and per time point

Figure 5A, B displays representative images of the graft of human ovarian tissue immunohistochemically stained for CD31 and CD34, respectively. Vessel density was evaluated by calculating CD31-positive vessels, H-MSCs + OT (107.52 ± 8.46) was significantly higher than OT and N-MSCs + OT groups (41.95 ± 6.76, vs. *P* < 0.001, 47.82 ± 7.93, vs. *P* < 0.001, respectively) on day 3. However, there was no significant difference between the three groups on day 7. The CD31-positive vessels on day 7 in three groups were significantly higher than on day 3 (*P* < 0.001, *P* < 0.001, *P* < 0.001, respectively).

Vessel density also was evaluated by calculating CD34-positive vessels. H-MSCs + OT (113.63 ± 8.29) was significantly higher than the OT group (73.93 ± 8.03, vs. *P* = 0.014) but not significantly higher than N-MSCs + OT groups (85.06 ± 8.68, vs. *P* = 0.252) on day 3. However, there were no significant differences between the three groups on day 7. The CD34-positive vessels on day 7 in three groups were significantly higher than on day 3 (*P* < 0.001, *P* < 0.001, *P* < 0.001, respectively).

### Follicle growth

A total of 160 primordial follicles were analysed for Ki67 (+) marker: 12, 20, and 27 from the OT, N-MSCs + OT, and H-MSCs + OT groups, respectively, on day 3, and 35, 39, and 27 from the OT, N-MSCs + OT, and H-MSCs + OT groups, respectively, on day 7. There were no significant differences between grafts (*P* = 0.756).

The growing follicle density in OT, N-MSCs + OT, H-MSCs + OT groups on day 3 was 31.25 ± 10.48, 81.07 ± 23.58, and 89.91 ± 30.87, respectively, and on day 7 was 62.19 ± 11.94, 65.05 ± 25.90, and 60.01 ± 24.36, respectively. There was no significant difference between the three groups and two-time points (*P* = 0.843).

### ROS and TAC

**Fig. 6:**
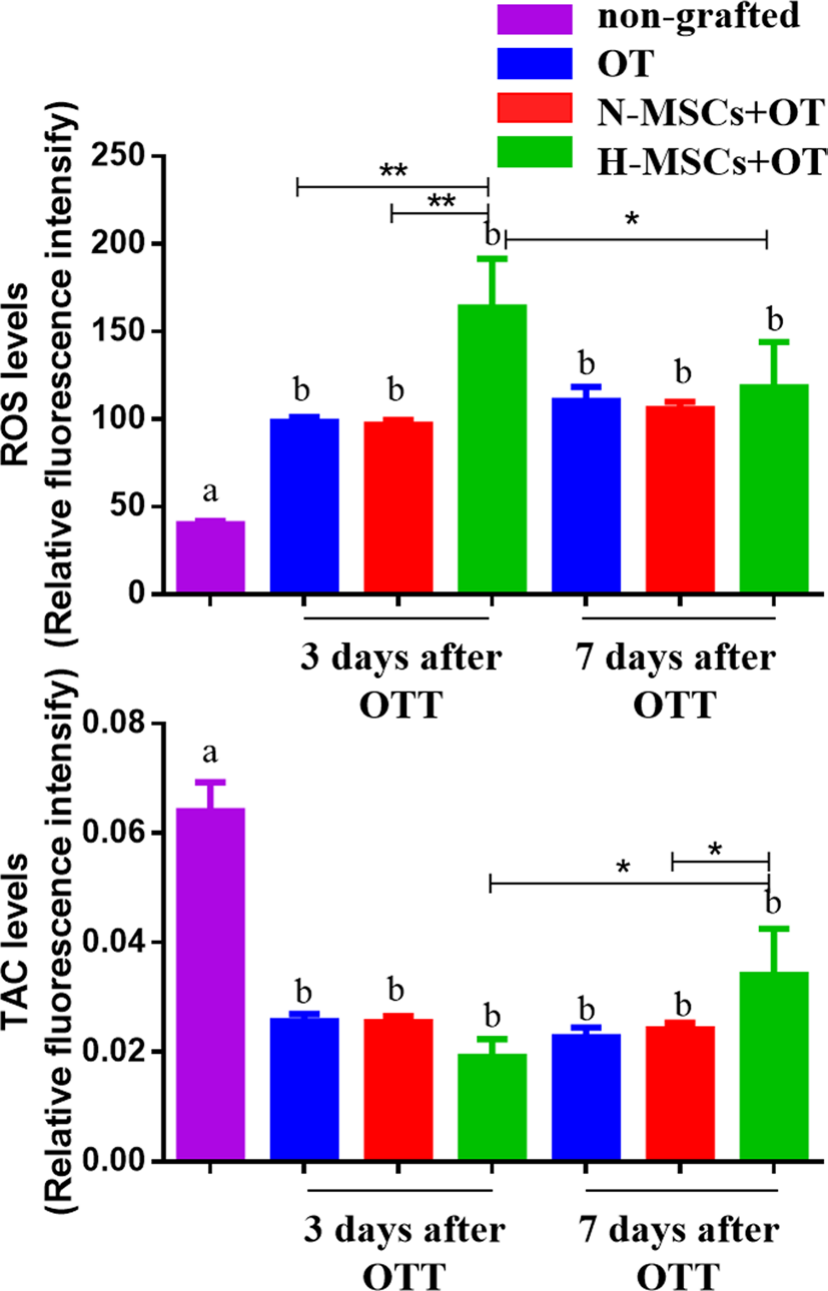
ROS and TAC measurement. Data were represented as mean ± SEM and analysed by one-way ANOVA and Fisher’s LSD post hoc test. *P* value references: *P < 0.05; **P < 0.01. Statistical significance is also expressed using different letters, a vs. b *P* < 0.05. ROS, reactive oxygen species; TAC, total antioxidant capability; OT, ovarian tissue transplantation; N-MSCs + OT, normoxic-treated HucMSCs, and ovarian tissue co-transplantation. H-MSCs + OT, hypoxia-treated HucMSCs, and ovarian tissue co-transplantation. *n* = 4 in the non-grafted group and *n* = 5/6 per grafted group and per time point

The ROS values in the non-grafted group were significantly lower than those in other grafts. The ROS values in the H-MSCs + OT (163.66 ± 27.89) group were significantly higher than the OT and N-MSCs + OT (98.43 ± 2.77, vs. *P* = 0.004, 96.87 ± 2.50, vs. *P* = 0.003, respectively) groups on day 3. There were no significant differences between the three groups on day 7 (Fig. 6). However, the ROS level of the H-MSCs + OT group on day 7 was significantly lower than day 3 (*P* = 0.039).

The TAC levels in the non-grafted group were significantly higher than those in other grafts. There were no significant differences in H-MSCs + OT (0.019 ± 0.003) compared with OT and N-MSCs + OT (0.026 ± 0.001, vs *P* = 0.305, 0.026 ± 0.001, vs *P* = 0.315, respectively) on day 3. The TAC levels in the H-MSCs + OT group (0.034 ± 0.008) were significantly higher than the OT group (0.023 ± 0.002, vs *P* = 0.049), but not N-MSCs + OT (0.024 ± 0.001, vs *P* = 0.122) on Day 7 (Fig. 6). The TAC level of the H-MSCs + OT group on day 7 was significantly higher than day 3 (*P* = 0.021).

### Western blot

**Fig. 7:**
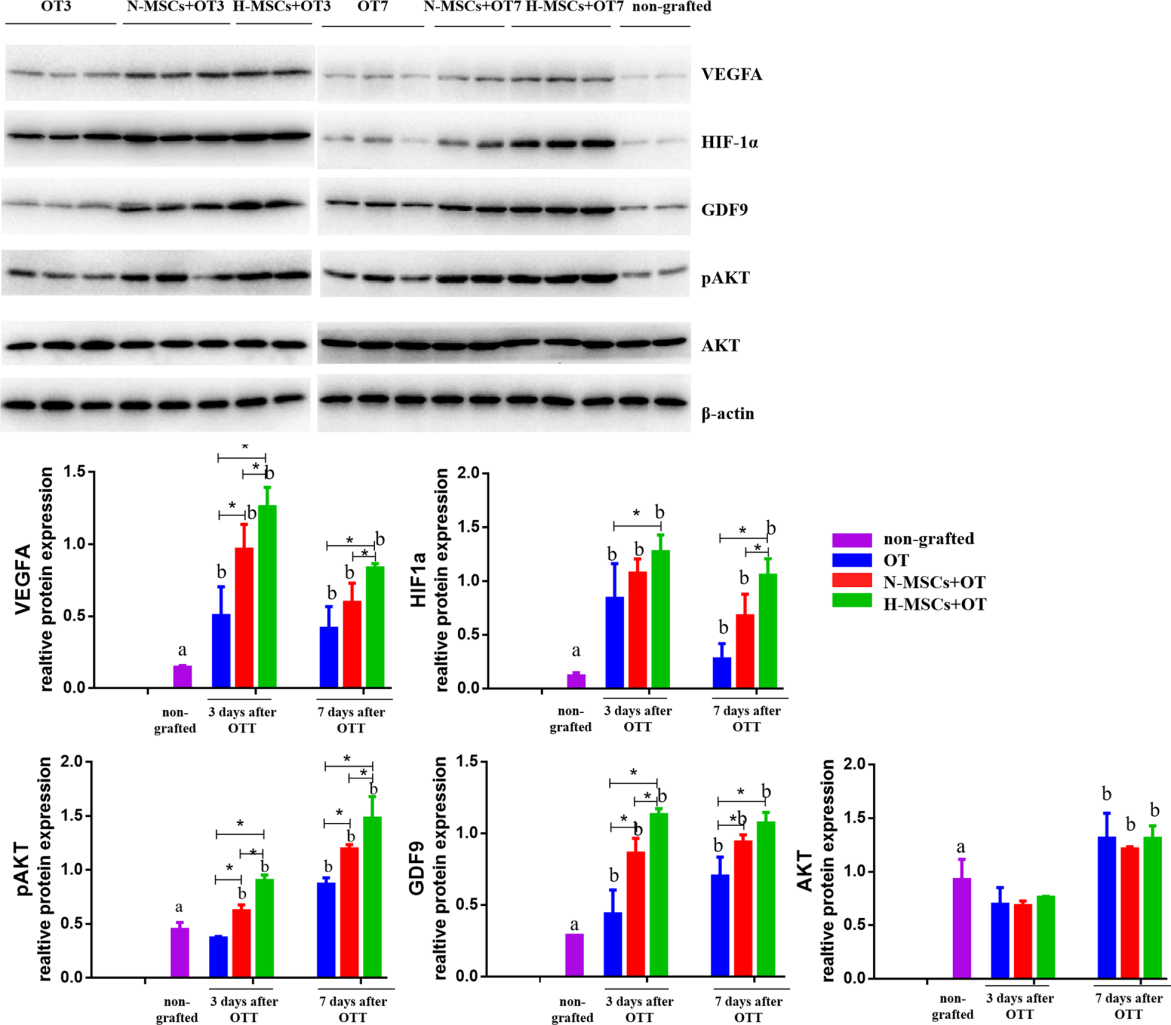
Relative protein expression of *VEGFA*, *HIF1α*, *GDF9*, *p-Akt*, and *Akt* genes in non-grafted and grafts. Data were represented as mean ± SEM and analysed by one-way ANOVA and Fisher’s LSD post hoc test. *P* value references: *P < 0.05. Statistical significance is also expressed using different letters, a vs. b *P* < 0.05. VEGFA, vascular endothelial growth factor A; HIF1α, hypoxia-inducible factor-1α; GDF9, growth differentiation factor 9; p-Akt, phosphor-protein kinase B; Akt, protein kinase B; OT, ovarian tissue transplantation; N-MSCs + OT, normoxic-treated HucMSCs and ovarian tissue co-transplantation; H-MSCs + OT, hypoxia-treated HucMSCs and ovarian tissue co-transplantation

The *HIF1α*, *VEGFA*, *pAkt*, *Akt*, and *GDF9* protein expressions were assessed in grafts. The grafts had a significantly higher expression of *HIF1a*, *VEGFA*, and *GDF9* than non-grafted. The grafts besides the OT group on day 3 had a higher expression of *pAkt* than non-grafted (Fig. 7).

*VEGFA* was significantly higher in the H-MSCs + OT group on day 3 than OT and N-MSCs + OT (*P* < 0.001, *P* = 0.045, respectively), and N-MSCs + OT was significantly higher than the OT group (*P* = 0.002). On day 7, the H-MSCs + OT group was significantly higher than OT and N-MSCs + OT (*P* = 0.004, *P* = 0.045, respectively).

*HIF1a* was significantly higher in the H-MSCs + OT group on day 3 than OT (*P* = 0.022). On day 7, the H-MSCs + OT group was significantly higher than OT and N-MSCs + OT (*P* = 0.000, *P* = 0.021, respectively).

Regarding *pAkt*, the H-MSCs + OT group on day 3 was significantly higher than OT and N-MSCs + OT (*P* < 0.001, *P* = 0.009, respectively), and N-MSCs + OT was significantly higher than the OT group (*P* = 0.008). On day 7, the H-MSCs + OT group was significantly higher than OT and N-MSCs + OT (*P* < 0.001, *P* = 0.008, respectively), and N-MSCs + OT was significantly higher than the OT group (*P* = 0.003).

Regarding *Akt*, on day 3, there were no differences in H-MSCs + OT compared with OT and N-MSCs + OT (*P* = 0.632, *P* = 0.559, respectively). On day 7, there was no difference in H-MSCs + OT compared with N-MSCs + OT and OT (*P* = 0.987, *P* = 0.463, respectively).

Regarding *GDF9*, on day 3, the H-MSCs + OT group was significantly higher than OT and N-MSCs + OT (*P* < 0.001, *P* = 0.019, respectively), and N-MSCs + OT was significantly higher than the OT group (*P* = 0.001). On day 7, the H-MSCs + OT group was significantly higher than OT (*P* = 0.001), and N-MSCs + OT was significantly higher than the OT group (*P* = 0.034).

### Hormone levels in mice' plasma

**Fig. 8:**
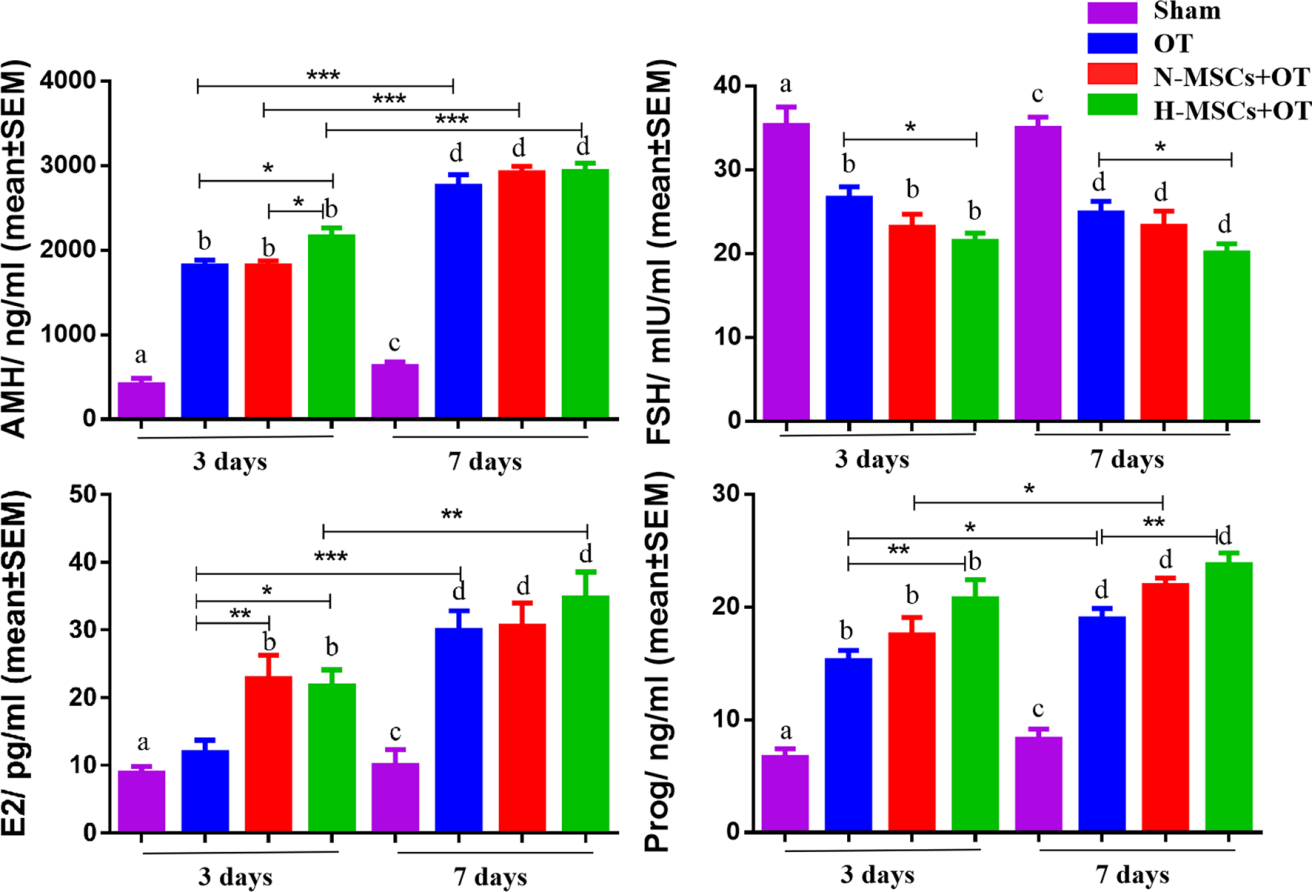
AMH, FSH, E2, and Prog concentration in mouse plasma. Data were represented as mean ± SEM and analysed by one-way ANOVA and Fisher’s LSD post hoc test. *P* value references: *P < 0.05; **P < 0.01; ***P < 0.001. Statistical significance is also expressed using different letters, a vs. b *P* < 0.05; c vs. d *P* < 0.05. AMH: anti-Müllerian hormone. FSH: follicle-stimulating hormone. E2: 17 β-estradiol. Prog: progesterone. Sham, sham-operated; OT, ovarian tissue transplantation; N-MSCs + OT, normoxic-treated HucMSCs, and ovarian tissue co-transplantation; H-MSCs + OT, hypoxia-treated HucMSCs, and ovarian tissue co-transplantation

After OT, the levels of AMH, E2, and Prog were increased, the FSH was decreased (Fig. 8). Regarding AMH, on day 3, the Sham (408.60 ± 76.82) group was significantly lower than OT, N-MSCs + OT, and H-MSCs + OT (1815.31 ± 68.13, vs. *P* < 0.001, 1814.29 ± 60.94, vs. *P* < 0.001, 2157.16 ± 109.01, vs. *P* < 0.001, respectively). On day 7, the Sham (626.01 ± 51.20) group was also significantly lower than OT, N-MSCs + OT, and H-MSCs + OT (2756.71 ± 139.34, vs. *P* < 0.001, 2917.65 ± 77.52, vs. *P* < 0.001, 2932.92 ± 98.03, vs. *P* < 0.001, respectively). On day 3, the H-MSCs + OT was significantly higher than OT and N-MSCs + OT (*P* = 0.022, *P* = 0.016, respectively) groups. However, on day 7, there were no significant differences between the three groups. On day 7, the AMH level in OT, N-MSCs + OT, and H-MSCs + OT three groups were significantly higher than on day 3 (*P* < 0.001, *P* < 0.001, *P* < 0.001, respectively).

Regarding FSH, on day 3, the Sham (35.30± 2.19) group was significantly higher than OT, N-MSCs + OT, and H-MSCs + OT (26.62 ± 68.13, vs. *P* < 0.001, 23.14 ± 1.56 vs. *P* < 0.001, 21.49 ± 0.96, vs. *P* < 0.001, respectively). On day 7, the Sham (34.95 ± 1.33) group was also significantly higher than OT, N-MSCs + OT, and H-MSCs + OT (24.87 ± 1.40, vs. *P* < 0.001, 23.27 ± 1.81, vs. *P* < 0.001, 20.07 ± 1.13, vs. *P* < 0.001, respectively). On day 3, the H-MSCs + OT was significantly lower than the OT group (*P* = 0.025) but not significantly lower than the N-MSCs + OT group (*P* = 0.477). On day 7, the H-MSCs + OT was significantly lower than the OT group (*P* = 0.039) but not significantly lower than the N-MSCs + OT group (*P* = 0.164).

Regarding E2, the Sham (8.87 ± 0.96) group on day 3 was significantly lower than N-MSCs + OT, and H-MSCs + OT (22.81 ± 3.46 vs. *P* < 0.01, 21.69 ± 2.43, vs. *P* < 0.01, respectively). On day 7, the Sham (9.98 ± 2.40) group was significantly lower than OT, N-MSCs + OT, and H-MSCs + OT (29.93 ± 2.88, vs. *P* < 0.001, 30.56 ± 3.43, vs. *P* < 0.001, 34.74 ± 3.81, vs. *P* < 0.001, respectively). On day 3, the N-MSCs + OT and H-MSCs + OT groups were significantly higher than the OT group (*P* = 0.009, *P* = 0.014, respectively). On day 7, the N-MSCs + OT and H-MSCs + OT groups were not significantly different from the OT group (*P* = 0.873, *P* = 0.221, respectively). On day 7, the E2 level in OT and H-MSCs + OT groups were significantly higher than on day 3 (*P* < 0.001, *P* = 0.001, respectively). The E2 level in the N-MSCs + OT group was non- significantly higher than on day 3 (*P* = 0.059, respectively).

Regarding Prog, on day 3, the Sham (6.66 ± 0.78) group was significantly lower than OT, N-MSCs + OT, and H-MSCs + OT (15.26 ± 0.89 vs. *P* < 0.001, 17.55 ± 1.54 vs. *P* < 0.001, 20.74 ± 1.70, vs. *P* < 0.001, respectively). On day 7, the Sham (8.30 ± 0.89) group was also significantly lower than OT, N-MSCs + OT, and H-MSCs + OT (18.96 ± 0.92, vs. *P* < 0.001, 21.92 ± 0.68, vs. *P* < 0.001, 23.79 ± 1.03, vs. *P* < 0.001, respectively). On day 3, the H-MSCs + OT was significantly higher than the OT group (*P* = 0.001) but not significantly lower than the N-MSCs + OT group (*P* = 0.051). On day 7, the H-MSCs + OT was significantly higher than the OT group (*P* = 0.0.005) but not significantly lower than the N-MSCs + OT group (*P* = 0.266). On day 7, the Prog level in OT, N-MSCs + OT groups were significantly higher than day 3 (*P* = 0.031, *P* = 0.014, respectively).

## Discussion

To our knowledge, this is the first study to investigate the effect of hypoxia-preconditioned HucMSCs on the human ovarian cortex in connection with OT. Although OT is an effective strategy for fertility preservation, the avascular nature of the grafting technique itself induces major stress in grafted tissue, with significant loss of the follicle reserve, which somewhat limits its potential and life span [16]. In this present study, we proved that hypoxia-preconditioned HucMSCs positively protect the grafted tissue by significantly decreasing the apoptosis and increasing higher expression of CD31, CD34, and VEGFA for earlier angiogenesis. They are crucial to preserving the resting primordial follicle pool by modulation of follicle death.

To reduce the loss of MSCs when MSCs and ovarian tissue were co-transplanted under the renal capsule of nude mice, thus affecting the appropriate evaluation of the effect of MSCs, we chose to co-transplant MSCs and Matrigel with ovarian tissue. At low temperatures, MSCs and Matrigel mixture is liquid. It then becomes solid after transplantation into nude mice, so that MSCs can be fixed around the ovarian tissue to exert their effect. MSCs mixed in Matrigel and transplanted with ovarian tissue have previously been used. In a previous study, no differences were found between OT and OT + Matrigel groups regarding microvascular density, VEGF, FGF, and the number of primordial follicles [18]. Because of these results, we have chosen not to include this control group.

Ovarian tissue is handled into cortical strips to be stored for a future orthotopic or heterotopic transplant using an avascular re-anastomosis surgical procedure [39]. A series of stressful events caused by hypoxia in grafts are complex and involve various players. Activation of signalling pathways in response to hypoxia and oxidative stress not only allows cells to adapt to this stress, changing cell metabolism and promoting survival but also triggers any subsequent tissue events, such as angiogenesis and tissue remodelling, which strive to reduce tissue injury [35].

Recently, efforts have been developed to improve follicle outcomes by boosting angiogenesis with growth factors or hormones or reducing oxidative stress-related harm with antioxidants [40]. Those potentially positive effects on follicle survival after OT have not identified a clear advantage of one strategy over another. They used MSCs, which have beneficial effects when applied to wound-healing models evaluating proangiogenic behaviour [41]. OT strategy was developed to improve follicle outcomes using adipose-derived MSCs (ASCs) to prepare the OT site before grafting [19, 42]. The ASCs could boost the vascularization of grafts. This strategy can decrease follicle loss that occurs soon after OT by reducing both follicle death and abnormal primordial follicle activation [35] and be persistent in the long term [16]. The umbilical cord is discarded after childbirth but compared to MSCs derived from traditional cell sources such as adipose or bone marrow, HucMSCs showed increased pluripotency and elevatory proliferation capacity [43]. Therefore, HucMSCs may be more suitable for cell therapy. However, MSCs were cultured at atmospheric oxygen tension, not representing their physiological conditions [22]. Therefore, low oxygen tension must be considered to cultivate MSCs to obtain a more accurate and reliable result [22]. Hypoxic preconditioning was protective against MSCs apoptosis induced by hypoxia and re-oxygenation via upregulation of *VEGF* and *Bcl-2*, a cellular protein that inhibits apoptosis and stabilizes mitochondrial membrane potential [44].

### Follicle survival and growth

This study evaluated follicle density with a particular investigation of the resting follicle pool. Preservation of the primordial follicle pool is crucial for ovarian tissue transplantation. The quantity and quality of primordial follicles are indeed major determinants, not only of fertility potential but also of graft lifespan. Our results showed that the follicular density of the H-MSCs + OT group is the highest, and the density of resting follicles is also the highest, which revealed that H-MSCs co-transplantation with ovarian tissue could preserve the survival of resting follicles. The follicle density in the N-MSCs group was lower than in the H-MSCs + OT group on 3 and 7 days of OT. There was no significant difference, meaning that N-MSCs were also protective of follicle survival, which is consistent with a series of studies on ASCs by Cacciottola et al. [16, 45].

The loss of primordial follicles during early ovarian tissue transplantation is still contentious. Indeed, two main mechanisms are proposed in the literature to explain the massive follicle loss seen after OT. One is direct follicle death due to hypoxia/ischaemia through apoptosis or necrosis [46]. The other is massive primordial follicle activation occurring due to numerous potential triggers of signalling pathways involved in follicle activation and growth [47, 48].

Regarding growing follicles, no upturn was observed after 3 or 7 days in any group. There was also no significant difference in the density of growing follicles between the three groups after 3 days and 7 days. Ki67 is a cell cycle-associated nucleoprotein antigen that serves as a proliferation marker during the active cell cycle stages (G1, S, G2, and mitosis) [19]. Ki67 staining correlates directly with activation of the flat granulosa cells of primordial follicles to cuboidal granulosa cells and follicular growth [49]. Our study investigated the percentage of Ki67 + primordial follicles in primordial follicles, and we did not find a significant difference between different groups and time points. These results may support follicle death as the major cause of hypoxia-related primordial follicle pool depletion.

### Stromal and follicle apoptosis

To address the hypothesis of follicle death as the major cause of depletion, we evaluated percentages of atretic follicles between groups over time. Follicle atresia is the resulting feature of several death processes. It occurs physiologically in follicle selection, but it also represents the outcome after apoptosis, necrosis, and non-apoptotic cell death processes [50]. In our study, significantly decreased graft stromal cell apoptosis and follicle were observed in the H-MSCs + OT group compared to the OT and N-MSCs + OT groups. Our results also showed that H-MSCs + OT has a lower percentage of atretic follicles and a lower percentage of TUNEL(+) follicles. Follicle atresia was evaluated using morphological parameters in HE-stained sections, including early and late signs of apoptosis. At the same time, TUNEL assays detected only extensive DNA degradation during the later stages of apoptosis [51].

These findings point to the hypoxia-treated HucMSCs in preventing large-scale follicle loss by decreasing follicle death, supporting the hypothesis that hypoxia-treated HucMSCs reduce follicle loss by decreasing hypoxia/ischaemia-related follicle death. Differences observed in our study may also be explained by the anti-apoptotic properties of hypoxia-preconditioned HucMSC acting via more paracrine secretion of VEGFA, basic fibroblast growth factor (bFGF), transforming growth factor β1 (TGFβ1), insulin-like growth factor 1 (IGF1), hepatocyte growth factor (HGF), etc. and by suppression of expression of inflammatory cytokines like BCL-2 modifying factor and tumour necrosis factor α (TNFα) [19, 26]. The growth factors VEGF, TGFβ, and bFGF, have well-characterized roles in vascular biology, where they can stimulate endothelial cells proliferation, sprouting, migration, and survival.

Another rational explanation for the observed decrease in apoptosis might be the earlier resumption of the O_2_ supply to the graft in the H-MSCs + OT group, which may have resulted in less severe hypoxia damage. Their anti-apoptotic impact could be due to direct results of the more secretome from hypoxia-preconditioned HucMSCs [52]. The secretome refers to the factors secreted from MSCs. These factors are released in soluble factors or packed into extracellular vesicles (EVs) as exosomes and micro-vesicles [26].

### Graft vascularization

Immunolabelling of the CD31 and CD34 antigen analysed on days 3 and 7 showed a significantly higher vascularized area in the H-MSCs + OT group on days 3. Survival of follicles in grafts depends on the rapid establishment of the blood supply, proangiogenic growth factors generated, and their harmony of the re-vascularization soon after OT [10, 53]. Angiogenesis represents a complex process that consists of multiple spatially and temporally regulated processes such as migration, sprout, tube formation, proliferation, and vascular and stromal remodelling. Regulation of ovarian angiogenesis in hypoxia is a complex process involving multiple vasoactive and angiogenic factors.

The main regulator of response to hypoxia is HIFs [54, 55]. HIFs are a family of DNA-binding transcription factors that regulate a series of hypoxia-related genes when cells respond to low oxygen conditions. In hypoxia, Akt is activated and plays a critical role in cell growth and survival [56]. Several mechanisms have been proposed for the protective effect of *HIF1α* [24]. Hypoxia-related responses enhance angiogenesis by un-regulating several growth factors. VEGF, an endothelial-specific mitogen and survival factor, is a powerful promoter of angiogenesis and neovascularization and is widely expressed in the ovary [13]. One study found that in endothelial cells, HIF1α is associated with acute cellular metabolic adaptations to rapid changes in O_2_ concentrations, whereas HIF2α mediates a wider role in tissue revascularization and regenerative processes [57]. However, we only focused on the evaluation of HIF1α in our study. In the future, we should also pay attention to the role of HIF2α in ovarian tissue transplantation.

In our study, the *HIF1α* of the ovary in the H-MSCs + OT group had a higher expression than in the OT and N-MSCs + OT groups, the tendency being in line with *VEGFA* which was one of the main effectors triggered by HIF1α signalling. A notable upturn of *VEGFA* was detected in both grafted groups 3 and 7 days after transplantation, consistent with the results of previous studies [58]. This higher hypoxia-independent expression of *VEGFA* in the H-MSCs + OT and N-MSCs + OT groups 3 and 7 days after transplantation may be because of the secretion of proangiogenic growth factors in the HucMSCs to ensure better revascularization. H-MSCs + OTs had higher *VEGFA* expression than N-MSCs + OTs after 3 and 7 days, which may be because of the more increased secretion of proangiogenic growth factors in hypoxia-preconditioned HucMSCs [26]. Its secretome is rich in various growth factors, including VEGF, governing the proangiogenic behaviour of HucMSC [29]. Boosting neoangiogenesis has proven to improve follicle outcomes [35]. Hypoxia-preconditioned HucMSCs provide prolonged exposure to growth factors such as VEGFA after transplantation, improving neoangiogenesis enhancement.

The *HIF1α* result differed from Cacciottola's study of *HIF1α* IHC staining in the primordial follicle, which found a lower expression of *HIF1α* 3 days after OT with ASCs [50]. They explained this by suggesting an earlier resumption of normoxic values. They found the widespread expression of *HIF1α* was detected in the follicles rather than in the stroma and irrespective of transplantation. We evaluated *HIF1α* levels in the whole ovarian cortex and grafts, which is different to primordial follicles. *HIF1α* may not directly but indirectly affect the follicles, such as enhanced revascularization [59]. On day 7, the H-MSCs + OT still had higher expression of *HIF1α*, maybe because the detected ovarian cortical tissues mixed with the hypoxic preconditioned HucMSCs was injected near the ovarian cortex. Using fluorescence labelling to evaluate the location and expression of HIF1α in stromal cells, primordial follicles, and growing follicles in ovarian cortical slices may better explain the effect of *HIF1α* on follicles.

The phosphatidylinositol-3 kinase (*PI3K*)/*Akt* signalling pathway has been shown to maintain primordial follicles [47, 60]. It is linked to hypoxia-related signalling, as *VEGF* also can trigger its activation for granulosa cell survival and proliferation [61]. Direct follicle death and massive primordial follicle activation were demonstrated in extensive investigations of follicle pool after OT, but to what extent is still unknown [62]. *PI3K*/*Akt* regulates a wide range of cellular functions, and it can also regulate the expression of *HIF1a* [24].

In this study, the *pAkt*, *HIF1a*, *VEGFA* was higher in grafts groups than the non-grafted group, the H-MSCs + OT group higher than OT, and N-MSCs + OT groups. However, the density of general and resting follicles was higher in the H-MSCs + OT group on day 3, but not with a significantly higher density of growing follicles. Another study found that the resting follicle pool in the ASC + OT group was better preserved. They explained this might be due to the shortened hypoxia period and therefore decreased hypoxia dependent *Akt* pathway activation in granulosa cells [19]. According to previous knowledge, the higher pAkt/HIF1α/VEGFA indicated more growing follicles in the H-MSCs + OT group. However, we found more resting follicles in the H-MSCs group, and the growing follicles were not significantly different to other grafts. This may be because the PI3K/Akt signalling pathway regulates a wide range of cellular functions and activates proteins associated with survival and inactivation of apoptosis-associated protein [24]. Our apoptosis results showed lower apoptosis of stromal cells and follicles in the H-MSCs + OT group. The *PI3K*/*Akt*/*HIF1a*/*VEGF*/*Akt* of signalling pathways is cyclic and complex with different backgrounds. We need to investigate *p-Akt* (+) or subcellular localization of Forkhead box O3 (FOXO3) in primordial follicles to understand the mechanism further. FOXO3 is known to play a critical role in maintaining the dormant state of primordial follicles. FOXO3 is regulated by phosphorylation via a PI3K-mediated signal. It is transported from the nucleus to the cytoplasm upon phosphorylation, thus triggering oocyte growth [63].

### ROS and TAC

Another possible explanation for the reduced apoptosis could be the earlier resumption of the O_2_ supply to the graft in the H-MSCs + OT group. We evaluated the ROS and TAC levels in the non-grafted ovarian tissue and grafts. The ROS generation kinetics during progressive revascularization of transplanted ovarian tissue has been evaluated by microdialysis, an established method used to assess metabolites in vivo without altering their levels. They concluded that grafted tissue undergoes anaerobic metabolism towards days 9, then a period of partial recovery of aerobic metabolism in days 10–17, followed by its complete restoration. Regarding ROS generation, there were two peaks on post-grafting days 10 and 17 [8]. ROS are mainly produced by mitochondria in the phosphorylated chain when oxygen supply is restored [64]. Any significant ROS upturn in hypoxic/ischaemic context results from increased oxygen levels [65].

The higher ROS level in the H-MSCs + OT group than OT and N-MSCs + OT group on day 3 may reflect earlier re-oxygenation in the H-MSC + OT group [50]. Cacciottola et al. found no significant antioxidant response in nuclear factor erythroid 2-related factor 2 (Nrf2) pathway or oxidative stress-related DNA damage. They concluded that oxidative stress does not play a significant role in follicle pool impairment after OT. They also found that ROS does not appear to be associated with large-scale follicle loss that occurs before complete tissue revascularization [8]. In our study, ROS levels in the H-MSCs + OT group on day 7 had significantly decreased compared to day 3. The higher TAC in the H-MSCs + OT group than OT on day 7 also reflects the better benefit of hypoxia-treated HucMSCs on the ovarian graft. This point should be considered when interpreting this result. We should directly use microdialysis equipment to evaluate the oxygen partial pressure in ovarian grafts in the future.

### Hormonal levels in mice

The E2, Prog, and AMH levels in the grafts group were significantly higher than those in the Sham operation group. The FSH level was significantly lower than that in the Sham operation group. E2 production is an important activity in follicles, as it results in positive regulation of follicle growth and axis stimulation, with consequent menstrual cycle resumption in patients who have undergone OT [3]. AMH is generated by granulosa cells of growing follicles [66]. Plasma AMH kinetics were similar over time in grafted groups. The tendency of AMH was the same as GDF9 expression, which is a marker of oocyte development quality [67]. The H-MSCs + OT group showed higher Prog and lower FSH than the OT group on day 3 and 7. The H-MSCs + OT group showed higher E2 and AMH than the OT group on day 3, but not on day 7. These findings may be suggested better hormone secretion in the hypoxia-preconditioned HucMSCs + OT group. The density of growing follicles in the H-MSCs + OT group was slightly but not significantly higher than the OT and N-MSCs + OT group, it may be can explain the H-MSCs + OT group did not showed higher E2 and AMH than the OT group on day 7. There were fewer atretic follicles and higher GDF9 in the H-MSCs + OT group, explaining the hormone results.

We acknowledge that there are some limitations to our study. The first is the heterogeneous follicle distribution encountered within and between small human ovarian cortex samples [68], but our findings are encouraging. Our results suggest that the proposed transplantation using hypoxia-treated HucMSCs is a promising step towards potentially solving the problem of the massive follicle loss after OT. Secondly, we did not evaluate the non-grafted tissue in detail. Thirdly, we did not directly assess the oxygenation/hypoxic situation in the grafts. Finally, a significant limitation to the study is that no longer-term xenotransplantations were performed to exclude that the treatment is a transient effect. Long-term xenotransplantations need to be performed, such as after 4–6 weeks, to confirm that more follicles have survived as an effect of the treatment.

## Conclusions

In conclusion, for the first time, we demonstrated that hypoxia-preconditioned HucMSCs co-transplantation with ovarian tissue resulted in early vascularization of ovarian tissue in the early post-grafting period, which correlates with increased follicle survival and reduced stromal and follicular apoptosis. The *HIF1α*/*VEGFA* signal pathways play an important role in elucidating the mechanisms of action of hypoxia-preconditioned MSCs in the context of ovarian tissue transplantation, with a view to clinical implementation.

## Data Availability

The data underlying this article are available in the article.

## Abbreviations

OTC: Ovarian tissue cryopreservation
OT: Ovarian tissue transplantation
ASCs: Adipose-derived mesenchymal stem cells
HucMSCs: Human umbilical cord mesenchymal stem cell
N-MSCs + OT: Normoxic-treated HucMSCs, and ovarian tissue co-transplantation
H-MSCs + OT: Hypoxia-treated HucMSCs, and ovarian tissue co-transplantation
POI: Premature ovarian insufficiency
ASRM: American Society for Reproductive Medicine
VEGF: Vascular endothelial growth factor
NAC: N-acetylcysteine
S1P: Sphingosine-1-phosphate
MSCs: Mesenchymal stem cells
HIF1α: Hypoxia-inducible factor 1α
SCID: Severe combined immunodeficient
HE: Haematoxylin and eosin
IHC: Immunohistochemistry
TUNEL: Terminal deoxynucleotidyl transferase dUTP nick-end labelling
WB: Western blot
ROS: Reactive oxygen species
TAC: Total antioxidant capacity
E2: 17β-Estradiol
FSH: Follicle-stimulating hormone
Prog: Progesterone
AMH: Anti-Müllerian hormone
GCs: Granulosa cells
DAPI: 4′,6-Diamidino-2-phenylindole
H2O2: Hydrogen peroxide
HRP: Horseradish peroxidase
DAB: 3,3′-Diaminobenzidine
DCHF-DA: 2′,7′-Dihydro-dichlorofluorescein diacetate
GDF9: Growth differentiation factor 9
p-Akt: Phosphor-protein kinase B
Akt: Protein kinase B
SDS-PAGE: Sulphate–polyacrylamide gel electrophoresis
PVDF: Polyvinylidene fluoride
SEM: Standard error of the mean
LSD: Least significant difference
bFGF: Basic fibroblast growth factor
TGFβ1: Transforming growth factor β1
IGF1: Insulin-like growth factor 1
HGF: Hepatocyte growth factor
TNFα: Tumour necrosis factor α
EVs: Extracellular vesicles
*PI3K*: Phosphatidylinositol-3 kinase
FOXO3: Forkhead box O3
Nrf2: Nuclear factor erythroid 2-related factor 2
